# A comparative analysis of masking empirical mode decomposition and a neural network with feed-forward and back propagation along with masking empirical mode decomposition to improve the classification performance for a reliable brain-computer interface

**DOI:** 10.3389/fncom.2022.1010770

**Published:** 2022-11-04

**Authors:** D. Jaipriya, K. C. Sriharipriya

**Affiliations:** School of Electronics Engineering, Vellore Institute of Technology, Vellore, India

**Keywords:** brain computer interface, signal processing, masking empirical mode decomposition, neural network with feed forward and back propagation, electroencephalography, motor imagery recognition

## Abstract

In general, extraction and classification are used in various fields like image processing, pattern recognition, signal processing, and so on. Extracting effective characteristics from raw electroencephalogram (EEG) signals is a crucial role of the brain-computer interface for motor imagery. Recently, there has been a great deal of focus on motor imagery in the EEG signals since they encode a person’s intent to do an action. Researchers have been using MI signals to assist paralyzed people and even move them on their own with certain equipment, like wheelchairs. As a result, proper decoding is an important step required for the interconnection of the brain and the computer. EEG decoding is a challenging process because of poor SNR, complexity, and other reasons. However, choosing an appropriate method to extract the features to improve the performance of motor imagery recognition is still a research hotspot. To extract the features of the EEG signal in the classification task, this paper proposes a Masking Empirical Mode Decomposition (MEMD) based Feed Forward Back Propagation Neural Network (MEMD-FFBPNN). The dataset consists of EEG signals which are first normalized using the minimax method and given as input to the MEMD to extract the features and then given to the FFBPNN to classify the tasks. The accuracy of the proposed method MEMD-FFBPNN has been measured using the confusion matrix, mean square error and which has been recorded up to 99.9%. Thus, the proposed method gives better accuracy than the other conventional methods.

## Introduction

BCI stands for brain-computer interaction and is a multidisciplinary as well as a multi-field interface between the human and the computer modality that includes computer science, neurology, the science of cognition and control, and medical science (Han et al., 2022). An information and command tunnel in the brain was established between the brain and the outside environment (Kevric and Subasi, 2017). Electroencephalography (EEG), a non-invasive scalp method, is a simple and low-cost way to capture brain activity. Multiple electrodes are implanted in certain scalp regions to record the EEG signal (Chan et al., 2015; Jin et al., 2020; Wolpaw et al., n.d.). The recent methods of an EEG signal include computed tomography, having temporal resolution with high resolution, even a millisecond. By this method, it is impossible to achieve a high resolution and this method also includes magnetic resonance imaging (Zhang et al., 2019; Hong et al., 2020). Because of these characteristics, EEG is a valuable tool for research and diagnosis in the field of brain function and diseases.

Motor imagery (MI) signals, one of the many different types of EEG signals, have recently attracted significant research interest since they are a relatively flexible EEG technique that allows us to distinguish between diverse brain activations (Lee and Choi, 2019; Ieracitano et al., 2021). When a person plans to move their hands or feet, their brain activity is recorded as motor imagery EEG. When you move your unilateral limb either left or right, the brain will change from an active state to an inactive state. Event-related synchronization (ERS) in the perception cortex will be mirrored in the cerebral motor, which includes event-related desynchronization (ERD) (Yuan et al., 2008). It is primarily manifested by an increase in the motor imagery cortex of the ipsilateral signal and a decrease in rhythms such as mu and beta (Abdalsalam et al., 2018) of the contralateral motor sensory cortex energy. The most basic physiological premise for motor imagery EEG classification is the one that is discussed above. As a result of these picturing or thinking tasks, the sensorimotor region of an EEG signal generates the motor imagery signal. The motor imagery signals have been used by various researchers to distinguish between various oscillatory brain activations for multiple tasks. Methods like machine learning as well as deep learning are used to achieve automated MI categorization (Ieracitano et al., 2021). Almost all classic studies have focused on two primary components: feature extraction and classification.

In the past, researchers used handmade features to identify EEG data using traditional techniques based on machine learning algorithms. Motor imagery signals have been used in BCI technology to create systems that use machine learning to assist stroke patients, including those with epilepsy, in communicating, controlling their wheelchairs and external devices, and more (Hramov et al., 2021). In addition to that, cognitive behavior as well as artificial intelligence will be used for EEG data in a deep understanding manner for human intelligent systems. However, because motor imagery signal’s spatial resolution and SNR will be very low, but the higher dynamic characteristics have a low spatial resolution, a low signal-to-noise ratio (SNR), and highly dynamic characteristics, extracting the crucial features will be a critical step in creating a brain-computer interface system (Schirrmeister et al., 2017). The main activity of classifying EEG signals is to analyze brain dynamics, which is a difficult undertaking due to these difficulties and the existence of enormous levels of noise in the data (Hernandez-Rojas et al., 2022; Hwaidi and Chen, 2022).

Traditional machine learning approaches have been successful in classifying motor imagery signals to some extent, but they were unsuccessful in their excellent decoding accuracy with customized features (Varsehi and Firoozabadi, 2021). Deep learning’s recent success has inspired academics to apply it to the signal classification of an EEG, and better results can be achieved for the extracted features, which can be extracted automatically by using deep learning techniques. Deep learning has produced results in several areas, including the classification of images and speech, and also detecting forgeries in various fields. From the signals, the stable spatial characteristics can be obtained by using convolutional neural networks (Lv et al., 2021). For applications such as video and audio classification, and to extract the temporal features to yield better results than the other models, recurrent neural networks has applied (Gong et al., 2022).

In the feature extraction methods, various domains are applicable such as frequency, time, time-frequency, and space-time-frequency. In added that, this can be classified as basic and advanced. The features of frequency and time are considered to be basic and the remaining two can be considered advanced (Lee et al., 2020; Bang et al., 2022). Combining time domain and frequency domain data, as well as adding spatial characteristics, can improve recognition ability. Furthermore, the neural network can adaptively extract EEG characteristics and a complete model can be achieved by the combination of extracted features into the classification of a signal. In the recognition of an EEG signal, two methods are used rapidly, exactly past 10 years. One is Linear Discriminant Analysis. Another one is the Support vector machine (Aggarwal and Chugh, 2019). To differentiate electroencephalogram data from the different interactions between the brain and the computer, a developed EEGNET and also DCNN are used for accurate evaluation. The result of the developed EEGNET has the best effect compared with the other algorithms used in the classification stage (Lawhern et al., 2018).

A combination of a CSP and CNN as a CSP-CNN achieves good accuracy on many motor imaging datasets (Yang et al., 2015). The classification training used the PSD of the signal as input, and the greater recognition rate was achieved by comprising both the characteristics of the time and frequency domains. In most cases, the characteristics of the frequency domain have been neglected (Tang Q. et al., 2022). Researchers suggested a new method for EEG categorization (Xu et al., 2019). Using the continuous wavelet transform (CWT), when compared to the STFT, the CWT has better time-frequency resolution. The images of the time-frequency have been used and can be obtained from the original electroencephalogram signals. Although the features of both time and frequency of an EEG have been used as an image instead of the signal by conversion, such images have lost some specific characteristics, which include the temporal and also the spatial. All the following research strategies manually extracted features using the usual feature extraction method. However, the only main issues with past investigations have been the low classification rate and also the poor SNR (Chaudhary et al., 2019; Tang Z. et al., 2022). It was challenging to achieve EEG end-to-end learning due to the above disadvantages.

**FIGURE 1:**
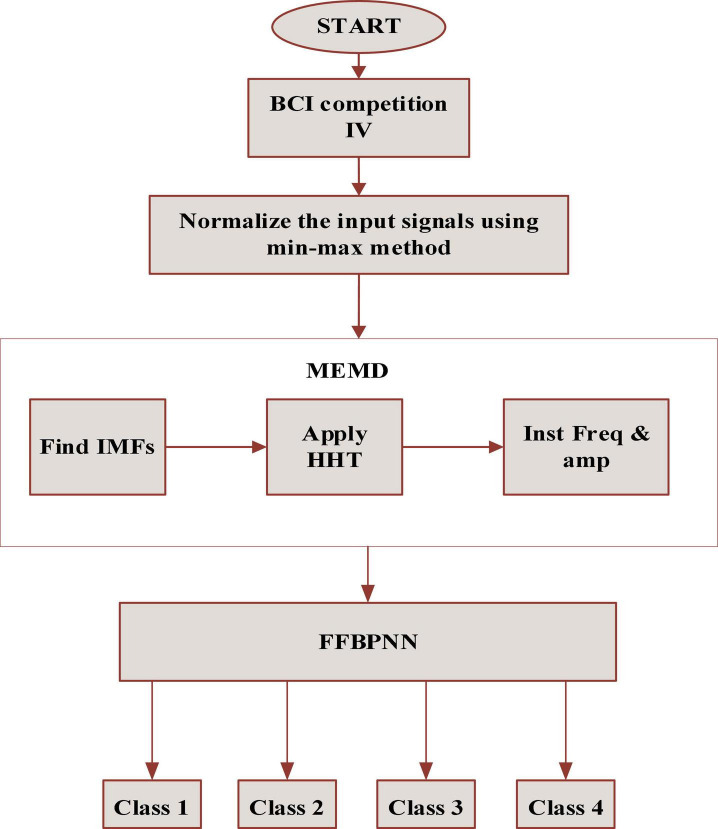
Flowchart of the proposed scheme.

The paper is organized as follows. In section “Related study,” we describe the motor imagery concept. In section “Materials and methods,” we describe the materials and techniques. In section “Proposed MEMD-FFBPNN method,” we describe the proposed method of masking empirical mode decomposition (MEMD)-based neural networks with feed-forward and backpropagation. In section “Results and discussion,” we describe the obtained results and the various comparison schemes with the existing methods, and in section “Conclusion,” we present the conclusion. The flowchart of the proposed scheme is shown in Figure 1.

## Related study

This section describes some features of the EEG signal and also the different machine learning techniques that have been used for feature extraction and classification.

### Motor imagery

For the decoding and extraction of MI signal features available in an EEG signal, various machine learning methods have been developed, and most of the methods are conventional.

Among the various approaches available for the extraction, a CSP-based approach of Filter bank common spatial patterns (Grosse-Wentrup and Buss, 2008; Ang et al., 2012) has produced the optimum outcomes. Many scientists have utilized many methods for classification, which include support vector machines (Rawashdeh et al., 2018). In noise removal, ICA and PCA methods are available, and these methods are also used for dimensionality reduction.

Multiple restricted Boltzmann machines (RBM) were employed to extract powerful features for the motor imagery dataset by researchers (Plis et al., 2014). For assessing spatial aspects and categorizing EEG signals, CNN has proven to be a popular choice (Huang and Wang, 2006; Cecotti and Gräser, 2011; Yang et al., 2015). As EEG recordings are time-series signals, DBN is used to extract the characteristics that are temporal (Yang et al., 2015; Lawhern et al., 2018; Zheng et al., 2020). Some studies show that CNN merged with RNN to extract the spatial as well as the temporal features (Huang and Wang, 2006; Cecotti and Gräser, 2011). In a study using EEG signals, CNN and autoencoders were employed to recognize emotions (Muhammad et al., 2017). Another study (Yang et al., 2015) used the short-time Fourier transform to turn the EEG information into pictures (STFT). Some researchers used mu and beta band characteristics for MI classification using CNN. In addition to that, the stacked autoencoder (SAE) is also used (Bengio et al., n.d.).

### Features of electroencephalography signal

In our proposed method, four different types of EEG signal features are covered in this paper. Such as energy, morphological features, fuzzy approximate entropy, and AR coefficients.

The sequence h (*n*) is written as follows:


(2.2.1) h⁢(n)={h⁢(1),h⁢(2),…,h⁢(N)}

The length of the signal is denoted as N.

### Energy

Energy has been considered as a significant measure of left-right MI EEG signal determination.


(2.2.2) $$E=\sum_{n=0}^{N-1}{\left|H_{t}\,\left(n\right)\right|}^{2}$$

### Morphological features

The considered EEG signal is denoted by X(t), and its morphological properties are the following: (Kalatzis et al., 2004):


(2.2.3) A⁢b⁢s⁢o⁢l⁢u⁢t⁢e⁢A⁢r⁢e⁢a⁢(A⁢A)=m⁢a⁢x⁢|x⁢(t)|

(2.2.4) $$P\,o\,s\,i\,t\,i\,v\,e\,A\,r\,e\,a\,\left(P\,A\right)=\sum_{t}0.5\times \left[x\,\left(t\right)+\left|x\,\left(t\right)\right|\right]$$

(2.2.5) $$N\,e\,g\,a\,t\,i\,v\,e\,A\,r\,e\,a\,\left(N\,A\right)=\sum_{t}0.5\times \left[x\,\left(t\right)-\left|x\,\left(t\right)\right|\right]$$

(2.2.6) T⁢o⁢t⁢a⁢l⁢A⁢r⁢e⁢a⁢(T⁢T)=P⁢A+N⁢A

(2.2.7) T⁢o⁢t⁢a⁢l⁢A⁢b⁢s⁢o⁢l⁢u⁢t⁢e⁢A⁢r⁢e⁢a⁢(T⁢A⁢A)=P⁢A+|N⁢A|

### Fuzzy approximate entropy

The following steps are used to get the fuzzy approximate entropy (FAP) (Pfurtscheller et al., 1998).

(A) The structure $X_{i}^{m}$ vector can be obtained by using a signal *x*(*i*)


(2.2.8) $$x_{i}^{m}=\left\{x\,\left(i\right),x\,\left(i+1\right),\ldots ,x\,\left(i+m-1\right)\right\}-x_{0}\,\left(i\right)$$

where i=1,  2,  …,  N–m+1

where,

*x*_*0*_(i) is defined below,


(2.2.9) $$x_{0}\,\left(i\right)=\frac{1}{m}\,\sum_{j=0}^{m-1}x\,\left(i+j\right)$$

where m is the sample length.

(B) To calculate the maximal distance $d_{i\,j}^{m}$, a fuzzy membership function has been decided by using the tolerance factor r.


(2.2.10) $$D_{i\,j}^{m}=e\,x\,p\,\left(-\frac{d_{i\,j}^{2}}{r}\right)$$

(C) The fuzzy approximate entropy given as follows:

The function Φ*^m^* is defined as,


(2.2.11) $$\Phi^{m}\,\left(n,r\right)=\frac{1}{N-m-1}\,\sum_{j=1,j\neq i}^{N-m+1}\ln C_{r}^{m}\,\left(i\right)$$

Where,

$C_{r}^{m}\,\left(i\right)$ is defined as the centroid of the fuzzy membership function and it is calculated by


(2.2.12) $$C_{r}^{m}\,\left(i\right)={\left(\text{N}-\text{m}+1\right)}^{-1}$$

Then,


(2.2.13) $$F\,A\,P\,\left(m,n,r,N\right)=\Phi^{m}\,\left(r\right)-\Phi^{m+1}\,\left(r\right)$$

### AR coefficients

AR models have been frequently employed in BCI research and have been shown and it has proven to be a useful feature in MI recognition tasks (Fang et al., 2015). The AR coefficients are used to represent the time-varying characteristics of signals (Zhou et al., 2008). Simultaneously, AR excels in modeling the EEG as filtered white noise with specific desired energy bands, making it ideal for EEG signal analysis, particularly for EEG signals. The Equation of the AR coefficient is given as,


(2.2.14) $$X\,\left(t\right)=\sum_{i=0}^{p}a\,\left(i\right)\,X\,\left(t-i\right)+e\,\left(t\right)$$

Added white noise is denoted as,

*e*(*t*). The correct Auto-regressive model order given as seven.

## Materials and methods

### Datasets

The BCI competition IV dataset 2a (Brunner et al., n.d.) are used in this paper. In the BCI competition IV, dataset 2a provides a 4-class motor imagery EEG signal given by the Knowledge Discovery Institute and the technology of Graz. The complete details of the datasets were described in the website of brain competition.

### Making empirical mode decomposition

Empirical Mode Decomposition (EMD), introduced by Huang, is a method for decomposing non-linear, multicomponent signals. The data-driven approach of empirical mode decomposition (EMD) decomposes a motor imagery EEG signal into a finite collection of band-limited functions known as intrinsic mode functions (IMFs). Each intrinsic mode function is the sequence of AM-FM frequency modulation. One of the intrinsic modes has been generated by using an EMD for signals along with an intermittent oscillation that can include the various kinds of wavelengths at distinct positions. A mode mixing problem occurs when these diverse oscillations exist at the same time, complicating analysis, and physical meanings that are not obvious. To mitigate the effects of mixing the modes, an algorithm is proposed, named as MEMD (Wang et al., 2014). In MEMD, an intrinsic mode function has been obtained from the input signal, and to obtain the frequency as well as the amplitude that is instantaneous, a method called the Hilbert transform is used (Deering and Kaiser, 2005).

### Feed forward back propagation neural network

In the brain-computer interface, an efficient system in the classification stage is employed in this work to classify two or more classes of motor imagery using supervised learning. The goal of categorization is to separate data from preprocessing into distinct categories. Furthermore, the EEG signal is recorded by the BCI system, and to reduce the error, the supervised learning algorithm has been used for the trained samples (Cecotti and Gräser, 2011; Cano-Izquierdo et al., 2012; Chai et al., 2014; Gandhi et al., 2014; Zhang et al., 2014; Ahirwal et al., 2016; Liu et al., 2016). Because of their capacity to learn implicitly, supervised learning algorithms are popular and to find intricate non-linear correlations of the variables with both dependent and independent components, and the possible interactions can be detected using independent variables between the predictor variables (Swetapadma and Yadav, 2015, 2016).

**FIGURE 2:**
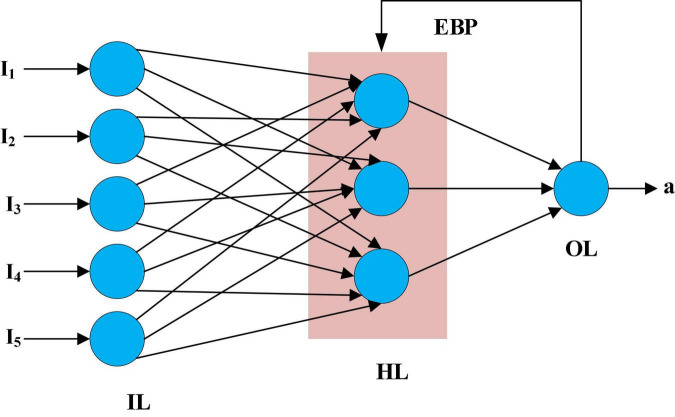
The structure of the Feed Forward Back Propagation Neural Network (FFBPNN).

Because of its capability to detect the correct patterns and classify the task, the neural network with feedforward and back-propagation has been used among several types of neural networks. The Widrow-Hoff learning algorithm is generalized to multiple-layer networks and non-linear differentiable transfer functions in a neural network with feedforward and back-propagation (FFBPNN). A network is trained using input vectors and their matching target vectors when the vectors of both the input and output functions have been matched approximately. Figure 2 depicts a simple FFBPNN with inputs, hidden layers, outputs, and so on. In Figure 2, I1 to I5 denote the input layer, HL denotes the hidden layer, OL denotes the output layer, EBP denotes the error backpropagation, and a denotes the overall output.

## Proposed MEMD-FFBPNN method

Our proposed method for MEMD-FFBPNN is composed of normalization using a min-max method, feature extraction using MEMD, and the feedforward back propagation neural network. In the first phase, the EEG data was preprocessed by normalization using the min-max method. Normalize the signals in the [+ 1, –1] range by using the Equation (4.1). The normalized data is denoted as, which has been described in the Equation below,


(4.1) $$Z_{i}=\frac{\left(y_{m\,a\,x}-y_{\min}\right)\times \left(x-x_{m\,i\,n}\right)}{\left(x_{m\,a\,x}-x_{m\,i\,n}\right)}+y_{m\,i\,n}$$

where *y*_*max*_ = + 1 and *y*_*min*_ = –1; x = (*x*_*1 ,      .    .    .   ,      x_n_*_)

where *i* = 1, 2,., *n*.

Then, the normalized signal is used to extract the features including the energy, fuzzy approximate entropy, AR coefficients, and morphological features using the MEMD. MEMD considers the decomposed signal as x(*n*), and the intrinsic mode functions have been obtained. The Hilbert Transform was then applied to the first IMF to extract the amplitude, *a*_*IMF1*_ and frequency *f*_*IMF*1_.

The instantaneous amplitude *a*_*z*_ can be obtained by using the instantaneous amplitude of the intrinsic mode function of the frequency masking signal given by,


(4.2) $$a_{z}=\frac{1.6}{N}\,\sum_{i=1}^{N}a_{I\,M\,F\,1}\,\,\left(i\right)$$

(4.3) $$f_{z}=\sum_{i=1}^{N}a_{I\,M\,F\,1}\,\,\left(i\right)\,f_{I\,M\,F}^{2}\,\left(\text{i}\right)\,/\sum_{i=1}^{N}a_{I\,M\,F\,1}\,\left(i\right)\,f_{I\,M\,F\,1}\,\left(i\right)$$

Then, the masking signal has been inserted into *x*(*n*), then the,


(4.4) x(n)+=x(n)+z(n)

(4.5) x(n)-=x(n)-z(n)

Thus, the above two sequences are obtained. These two sequences were used to obtain the IMFs, *y*^+^(n) of *x*^+^(n) as well as *y*^−^(n) of *x*^−^(n) by performing EMD. At last, the IMF *y*(*n*) of *x*(*n*) using MEMD as calculated and the Final MEMD output signal has been obtained by using the below Equation,


(4.6) $$y\,\left(n\right)=\left(y^{+}\,\left(n\right)+y^{-}\,\left(n\right)\right)/2$$

And then, finally, the extracted features are fed into the classifier. Here we used a feedforward neural network using backpropagation for classification. The following three steps are the feedforward neural network with backpropagation.

The input of the signal must propagate forward (Demuth and de Jesús, n.d.), which is the first and foremost step in FFBPNN, and the output is calculated by using the Equation (4.4),


(4.7) $$a^{n+1}=f^{n+1}\,\left(W^{n+1}\,a^{n+1}+b^{n+1}\right)$$

n = 0, 1, …, L – 1

where a is network output

f is the transfer function

Weight of the several layers is denoted as W

Bias matrices of several layers is denoted as B

In the back propagation procedure, the network’s backward propagation of the sensitivity begins with the bottom layer.


(4.8) $$s^{L}=-2\,F^{L}\,\left(x^{L}\right)\,\left(t-a\right)$$

where s is the sensitivity and *FL* is described as in Jana et al. (2018).

x is the net input

t is the target


(4.9) $$s^{n}=F^{n}\,\left(x^{n}\right)\,{\left(W^{n+1}\right)}^{T}\,s^{n+1}$$

In the final phase of the back propagation procedure, the weight has been updated using a steepest decent condition.


(4.10) $$W^{n}\,\left(k+1\right)=W^{n}\,\left(k\right)-\alpha \,s^{n}\,{\left(a^{n-1}\right)}^{T}$$

where the rate of the learning is denoted as α.

the final phase of the back propagation, biases can be updated by using the Equation 4.9.


(4.11) $$b^{n}\,\left(k+1\right)=b^{n}\,\left(k\right)-\alpha s^{n}$$

Then, we measured an error rate and accuracy of the classification at the end of the classifier.

**FIGURE 3:**
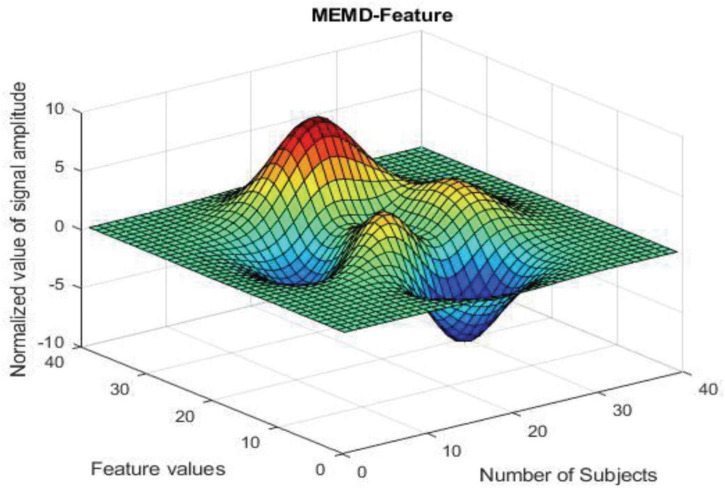
MEMD feature.

**FIGURE 4:**
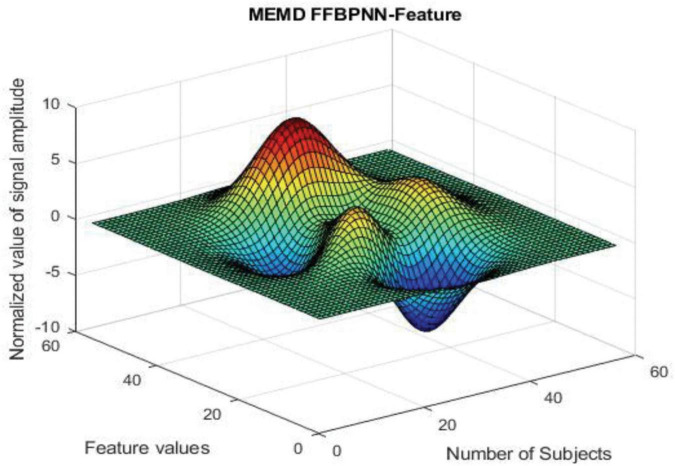
MEMD-FFBPNN feature.

**FIGURE 5:**
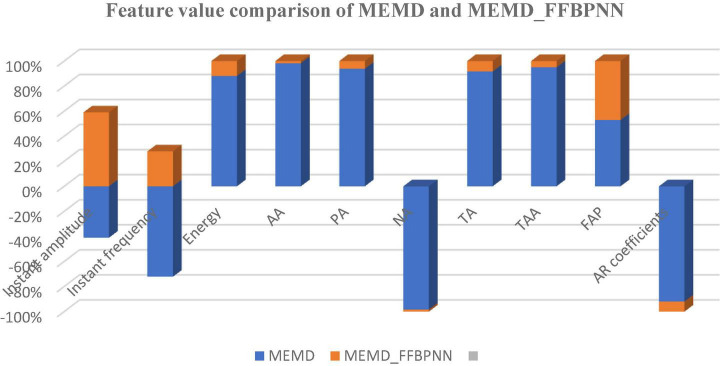
Feature value comparison of MEMD and MEMD-FFBPNN.

**FIGURE 6:**
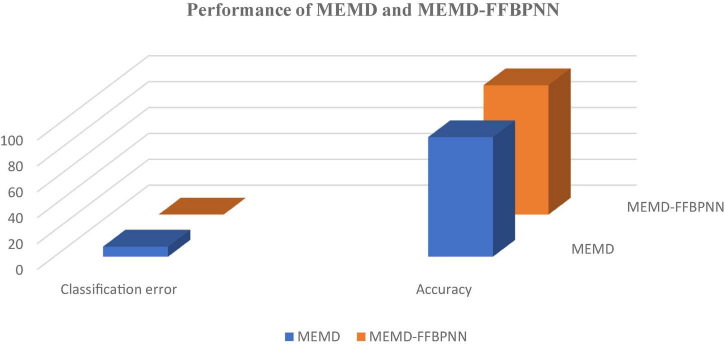
Comparison of the performance of MEMD and MEMD-FFBPNN.

MATLAB has been used for each algorithm’s creation and signal processing. The Figure 3 shows that the results in 3-dimensional structure (i.e.) Normalized value of signal amplitude, feature values and the number of subjects of the feature extraction method— MEMD. The Figure 4 shows that the results in 3-dimensional structure (i.e.) Normalized value of signal amplitude, feature values and the number of subjects of the feature extraction as well as the classification method— MEMD—Feed Forward Back Propagation Neural Network. The Figure 5 shows that the performance of the feature comparison of both MEMD and MEMD_FFBPNN, where the features include energy, morphological features, fuzzy approximate entropy, and AR coefficient values. The Figure 6 shows the performance comparison of both MEMD and MEMD_FFBP. Here, classification accuracy and classification error are the parameters taken. The classification accuracy of 92.28 and 99.94% achieved in MEMD and MEMD_FFBPNN, respectively. The classification error of 7.72 and 0.06% achieved in MEMD and MEMD_FFBPNN, respectively.

## Results and discussion

### Performance evaluation of the MEMD-FFBPNN technique with variable error rate

The motor imagery signal has been tested with the MEMD-FFBPNN technique by varying the error rate (mean square error). The analysis of the MEMD-FFBPNN is shown in Table 1 varying the error goals 10^–1^, 10^–2^, 10^–3^, and 10^–4^. The proposed method yields 99.9% accuracy with an error goal of 10^–2^. Thus, the proposed MEMD-FFBPNN method can more accurately categorize the motor imagery signals.

**TABLE 1:** Performance evaluation of the MEMD-FFBPNN technique with variable error rate.

Sl. No.	Error rate	Accuracy
1	10^–1^	96%
2	10^–2^	99.9%
3	10^–3^	98.2%
4	10^–4^	97.8%

### Performance evaluation of the MEMD-FFBPNN using transfer function

Various transfer functions are examined with the MEMD-FFBPNN for the performance evaluation. Performances of the MEMD-FFBPNN for various transfer functions, including purelin, log-sig, tan-sig, etc., are shown in Table 2. When a network is trained with a tan-sig transfer function, testing accuracy is improved. As a result, the neural network architecture using the MEMD-FFBPNN uses the tan-sig transfer function.

**TABLE 2:** Performance evaluation of the MEMD-FFBPNN using transfer function.

Sl. No.	Transfer function	Performance	
		
		Accuracy	Error rate
1	Purelin	96.7%	3.3%
2	Log-sig	98.4%	1.6%
3	Tan-sig	99.9%	0.057%

### Performance evaluation of the MEMD-FFBPNN using and excluding normalized input

The motor imagery signal has been tested both with and without normalized input, using the proposed MEMD-FFBPNN. Evaluation of the MEMD-FFBPNN using and excluding the normalized input is shown in Table 3. When a network is trained with normalized input as opposed to unnormalized input, testing accuracy is higher. Thus, the neural network design using the proposed MEMD-FFBPNN uses the normalized input.

**TABLE 3:** Performed evaluation of the MEMD-FFBPNN using and excluding normalized input.

Sl. No.	Input	Performance evaluation	
		
		Accuracy	Error rate
1	Excluding normalized	90.4%	9.6%
2	Normalized with min-max method	99.9%	0.057%

### Comparative analysis with the other works

**FIGURE 7:**
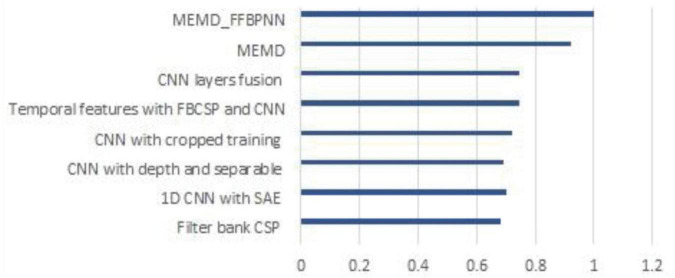
Performance of the existing methods and the proposed method.

In this proposed work, the dataset of the BCI competition IV dataset has been classified which is used for the motor imagery EEG signal and in their earlier work (Ang et al., 2012; Tabar and Halici, 2017; Sakhavi et al., 2018; Amin et al., 2019), many researchers classified the dataset of the BCI competition IV. Table 4 compares the proposed MEMD-FFBPNN’s accuracy to that of the competing approaches. Because of its simplicity and efficiency, the proposed algorithm has acquired better accuracy and a decreased classification error rate, which is also illustrated in Figure 7.

**TABLE 4:** Classification results obtained for the BCI dataset.

Methods	Accuracy
Filter bank CSP (Ang et al., 2012)	68.0%
1D CNN with SAE (Tabar and Halici, 2017)	70.0%
CNN with depth and separable (Lawhern et al., 2018)	69.0%
CNN with cropped training (Schirrmeister et al., 2017)	72.0%
Temporal features with FBCSP and CNN (Sakhavi et al., 2018)	74.4%
CNN layers fusion (Amin et al., 2019)	74.5%
MEMD	92.28%
MEMD_FFBPNN	99.94%

## Conclusion

The brain-computer interface (BCI) was created to connect behavioral and clinical theories. This introduction leads to the study of learning a new perspective on the classification of cognitive states in people and computers that considers the unique skills of the human brain for processing motor imagery signals. Soft computing, on the other hand, is a collection of approaches that aid in the conversion of the categorization of the cognitive state described above into a firm prediction. The proposed work provides a motor imagery categorization of the decomposition method, a MEMD, and a supervised learning system based on the neural network with feed-forward and backpropagation. This paper includes the comparison of the other existing methods with the proposed method and the performance of the proposed method by varying the error goals, and transfer function, with and without normalized inputs. This work mainly focused on the classification of the motor imagery signals using the MEMD along with feedforward backpropagation neural networks and achieved the highest accuracy of 99.9%.

Additionally, we anticipate that the findings in this paper may motivate other researchers to apply neural networks to enhance the brain signals to classify. Future research will focus on improving deep learning algorithms to better classify motor imagery EEG signals and create a more reliable interaction between the brain and the computer.

## Data availability statement

The original contributions presented in this study are included in the article/supplementary material, further inquiries can be directed to the corresponding author/s.

## Author contributions

Both authors contributed to content preparations and validation of context.
